# The genome sequence of a stiletto fly, *Thereva unica *(Harris, 1780)

**DOI:** 10.12688/wellcomeopenres.20828.1

**Published:** 2024-02-12

**Authors:** Martin Drake, Chris Spilling, Natural History Museum Genome Acquisition Lab, Darwin Tree of Life Barcoding collective, Wellcome Sanger Institute Tree of Life Management, Samples and Laboratory team, Wellcome Sanger Institute Scientific Operations: Sequencing Operations, Wellcome Sanger Institute Tree of Life Core Informatics team, Tree of Life Core Informatics collective, Darwin Tree of Life Consortium

**Affiliations:** 1 Independent researcher, Burridge, England, UK; 2 Natural History Museum, London, England, UK

**Keywords:** Thereva unica, a stiletto fly, genome sequence, chromosomal, Diptera

## Abstract

We present a genome assembly from an individual female *Thereva unica* (a stiletto fly; Arthropoda; Insecta; Diptera; Therevidae). The genome sequence is 910.1 megabases in span. Most of the assembly is scaffolded into 6 chromosomal pseudomolecules. The mitochondrial genome has also been assembled and is 17.66 kilobases in length.

## Species taxonomy

Eukaryota; Metazoa; Eumetazoa; Bilateria; Protostomia; Ecdysozoa; Panarthropoda; Arthropoda; Mandibulata; Pancrustacea; Hexapoda; Insecta; Dicondylia; Pterygota; Neoptera; Endopterygota; Diptera; Brachycera; Muscomorpha; Asiloidea; Therevidae; Therevinae; Thereva ( Harris, 1780) (NCBI:txid2867258).

## Background

*Thereva unica* ( Harris, 1780) is a member of the Therevidae (Diptera), known as stiletto flies on account of their conical abdomen. Short pubescence covers most of the body and in some species is brilliant silver but in *T. unica* is drab brown. This species was long known in Britain as *T. bipunctata* Meigen, 1820 as there was uncertainty over the identity of Harris’s species ( Chandler, 1998). His painting is unhelpful, but his description of the female’s frons with its two shining black spots is accurate ( Harris, 1780) and is similar only in occasional specimens of two rare British species and of the common *T. nobilitata* (Fabricius). On balance it seems likely that Harris’s specimen was the same as Meigen’s *T. bipunctata*. Species of *Thereva* can be difficult to name accurately, so the genome will help clarify the taxonomy. Larvae of *Thereva* cannot be identified using morphology so genomic identification will aid ecological research of this life stage.

*Thereva unica* is found on fixed dunes on the coast from Cumbria to Yorkshire, with disjunct populations in Scotland, including the Outer Hebrides. Inland it is found in dry sandy heaths in the Surrey area, the Breckland of East Anglia and isolated heaths elsewhere in England.

Therevid larvae are long, thin, and fairly featureless, but are remarkable for possessing a smooth dry cuticle which aids their ‘swimming’ through particulate substrates, rather like a stiff eel. They are hunting predators, detecting their prey by its vibrations and subduing it rapidly with a venom ( Smith, 1989; Stubbs & Drake, 2014). They are probably unspecific in their choice of prey, taking any other arthropods and worms although Irwin and Lyneborg (1981) state that beetle larvae are preferred. In related species living in dry sand, prey captured on the surface is dragged back into the sand (MD, pers. obs.). The feeding behaviour of adults is unclear but males of some species of *Thereva* swarm, so an energy intake would be necessary.

## Genome sequence report

**Figure 1.**
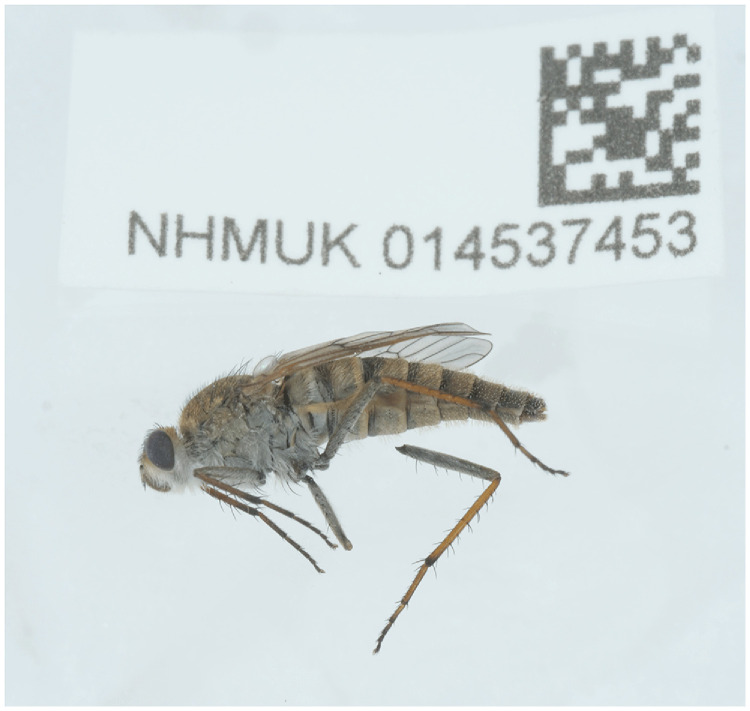
Photograph of the *Thereva unica* (idTheUnic2) specimen used for genome sequencing.

The genome was sequenced from one female *Thereva unica* ( Figure 1) collected from Loe Valley (River Cober), England, UK (50.09, –5.29). A total of 36-fold coverage in Pacific Biosciences single-molecule HiFi long reads was generated. Primary assembly contigs were scaffolded with chromosome conformation Hi-C data. Manual assembly curation corrected 249 missing joins or mis-joins and removed 48 haplotypic duplications, reducing the assembly length by 2.35% and the scaffold number by 18.25%, and increasing the scaffold N50 by 70.63%.

**Figure 2.**
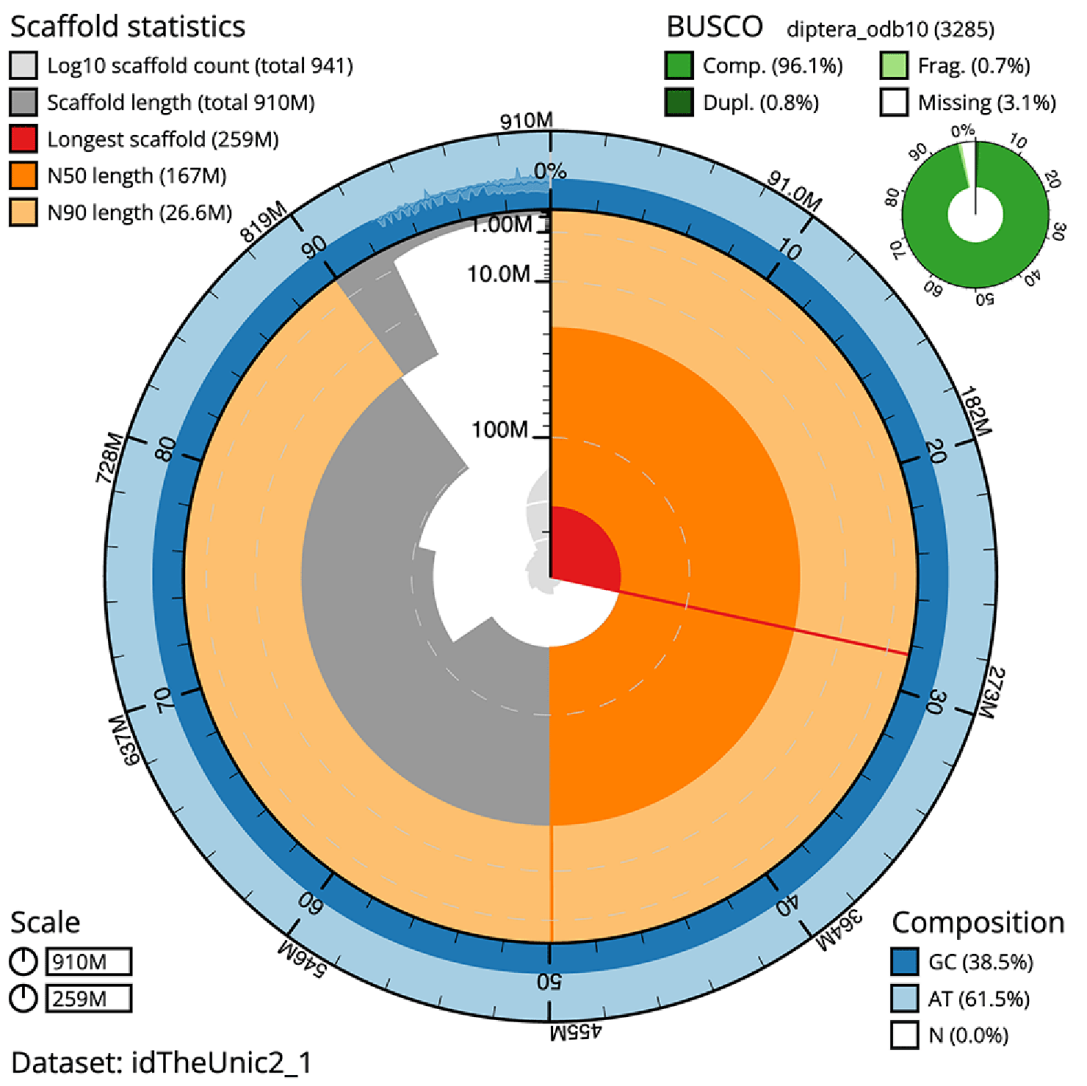
Genome assembly of *Thereva unica*, idTheUnic2.1: metrics. The BlobToolKit Snailplot shows N50 metrics and BUSCO gene completeness. The main plot is divided into 1,000 size-ordered bins around the circumference with each bin representing 0.1% of the 910,162,612 bp assembly. The distribution of scaffold lengths is shown in dark grey with the plot radius scaled to the longest scaffold present in the assembly (258,850,354 bp, shown in red). Orange and pale-orange arcs show the N50 and N90 scaffold lengths (167,368,244 and 26,588,518 bp), respectively. The pale grey spiral shows the cumulative scaffold count on a log scale with white scale lines showing successive orders of magnitude. The blue and pale-blue area around the outside of the plot shows the distribution of GC, AT and N percentages in the same bins as the inner plot. A summary of complete, fragmented, duplicated and missing BUSCO genes in the diptera_odb10 set is shown in the top right. An interactive version of this figure is available at https://blobtoolkit.genomehubs.org/view/idTheUnic2_1/dataset/idTheUnic2_1/snail.

**Figure 3.**
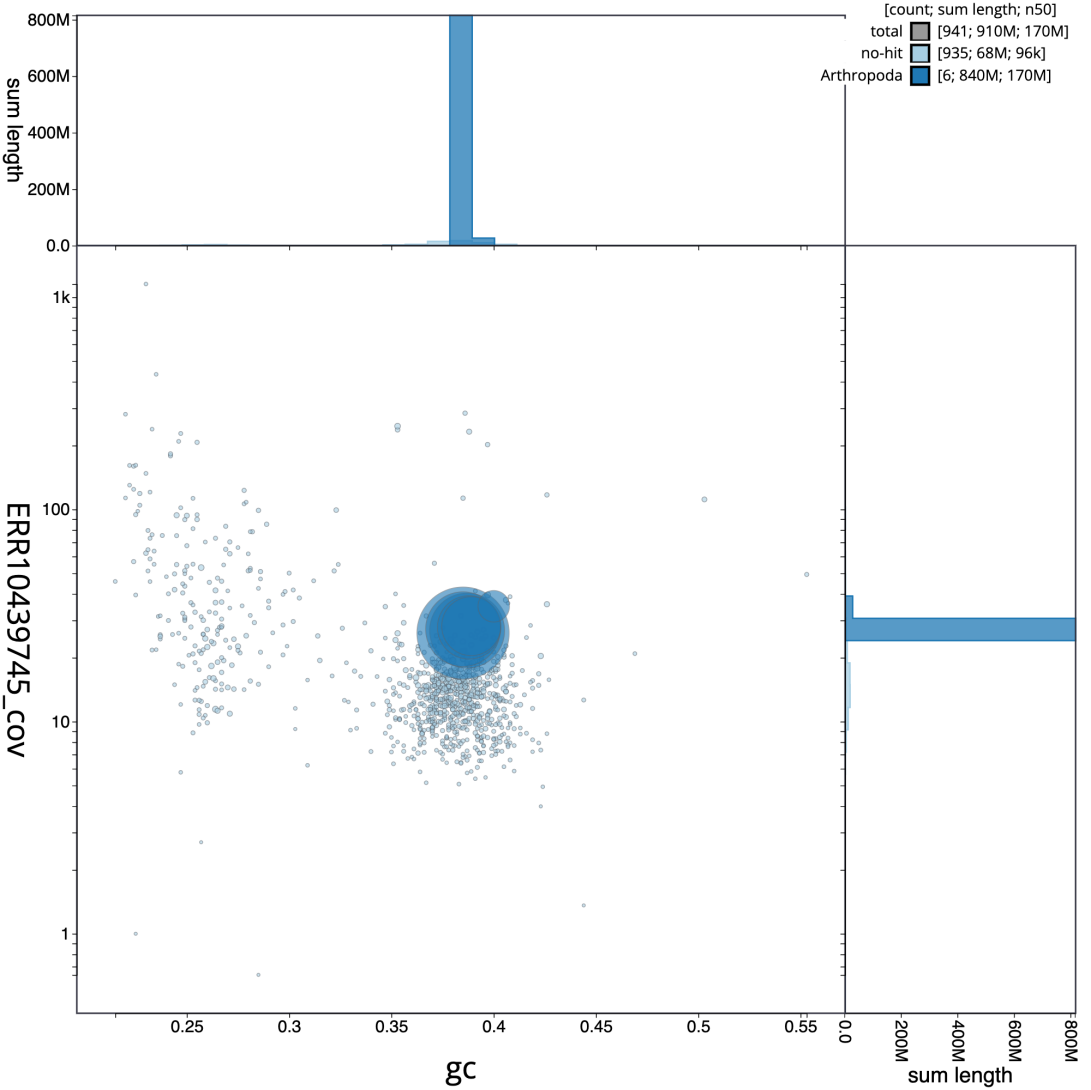
Genome assembly of *Thereva unica*, idTheUnic2.1: BlobToolKit GC-coverage plot. Scaffolds are coloured by phylum. Circles are sized in proportion to scaffold length. Histograms show the distribution of scaffold length sum along each axis. An interactive version of this figure is available at https://blobtoolkit.genomehubs.org/view/idTheUnic2_1/dataset/idTheUnic2_1/blob.

**Figure 4.**
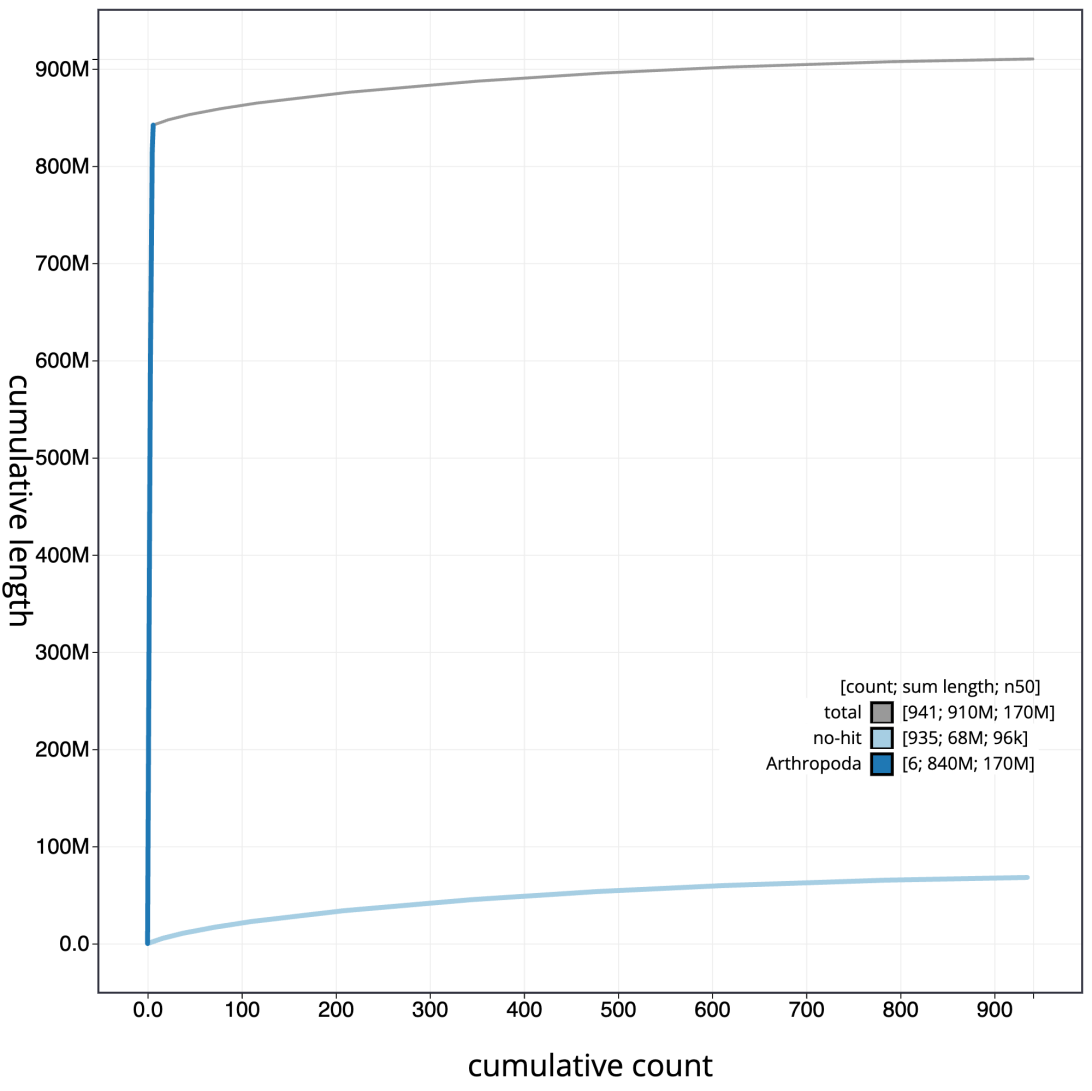
Genome assembly of *Thereva unica*, idTheUnic2.1: BlobToolKit cumulative sequence plot. The grey line shows cumulative length for all scaffolds. Coloured lines show cumulative lengths of scaffolds assigned to each phylum using the buscogenes taxrule. An interactive version of this figure is available at https://blobtoolkit.genomehubs.org/view/idTheUnic2_1/dataset/idTheUnic2_1/cumulative.

**Figure 5.**
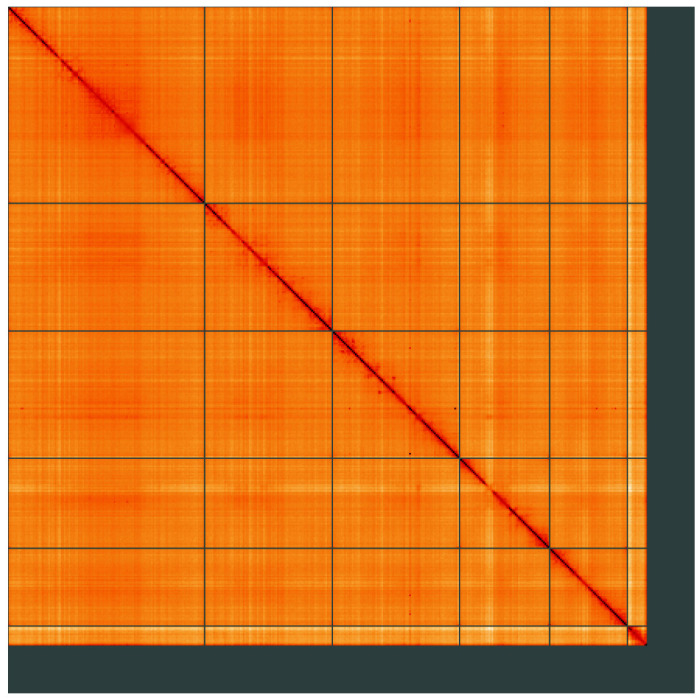
Genome assembly of *Thereva unica*, idTheUnic2.1: Hi-C contact map of the idTheUnic2.1 assembly, visualised using HiGlass. Chromosomes are shown in order of size from left to right and top to bottom. An interactive version of this figure may be viewed at https://genome-note-higlass.tol.sanger.ac.uk/l/?d=IU_qBrpWToWNrAiItglZPA.

The final assembly has a total length of 910.1 Mb in 940 sequence scaffolds with a scaffold N50 of 167.4 Mb ( Table 1). The snailplot in Figure 2 provides a summary of the assembly statistics, while the distribution of assembly scaffolds on GC proportion and coverage is shown in Figure 3. The cumulative assembly plot in Figure 4 shows curves for subsets of scaffolds assigned to different phyla. Most (92.52%) of the assembly sequence was assigned to 6 chromosomal-level scaffolds, representing 6 autosomes. Chromosome-scale scaffolds confirmed by the Hi-C data are named in order of size ( Figure 5; Table 2). The exact order and orientation of the scaffolds in the repetitive centromeres is unknown. As it is a female XX sample without a comparator species, the X chromosome is unidentified. While not fully phased, the assembly deposited is of one haplotype. Contigs corresponding to the second haplotype have also been deposited. The mitochondrial genome was also assembled and can be found as a contig within the multifasta file of the genome submission.

**Table 1.** Genome data for *Thereva unica*, idTheUnic2.1.

Project accession data		
Assembly identifier	idTheUnic2.1	
Species	*Thereva unica*	
Specimen	idTheUnic2	
NCBI taxonomy ID	2867258	
BioProject	PRJEB57101	
BioSample ID	SAMEA110026478	
Isolate information	idTheUnic2, female: whole organism (DNA sequencing) idTheUnic1, male: head and thorax (HiC sequencing)	
Assembly metrics*		*Benchmark*
Consensus quality (QV)	58.8	*≥ 50*
*k*-mer completeness	99.99%	*≥ 95%*
BUSCO**	C:96.1%[S:95.3%,D:0.8%], F:0.7%,M:3.1%,n:3,285	*C ≥ 95%*
Percentage of assembly mapped to chromosomes	92.52%	*≥ 95%*
Sex chromosomes	Not identified	*localised* *homologous pairs*
Organelles	Mitochondrial genome: 17.66 kb	*complete single* *alleles*
Raw data accessions		
PacificBiosciences SEQUEL II	ERR10439745	
Hi-C Illumina	ERR10446380	
Genome assembly		
Assembly accession	GCA_949987705.1	
*Accession of alternate* *haplotype*	GCA_949987715.1	
Span (Mb)	910.1	
Number of contigs	1865	
Contig N50 length (Mb)	2.2	
Number of scaffolds	940	
Scaffold N50 length (Mb)	167.4	
Longest scaffold (Mb)	258.85	

* Assembly metric benchmarks are adapted from column VGP-2020 of “Table 1: Proposed standards and metrics for defining genome assembly quality” from ( Rhie *et al.*, 2021). ** BUSCO scores based on the diptera_odb10 BUSCO set using version 5.3.2. C = complete [S = single copy, D = duplicated], F = fragmented, M = missing, n = number of orthologues in comparison. A full set of BUSCO scores is available at https://blobtoolkit.genomehubs.org/view/idTheUnic2_1/dataset/idTheUnic2_1/busco.

**Table 2.** Chromosomal pseudomolecules in the genome assembly of *Thereva unica*, idTheUnic2.

INSDC accession	Chromosome	Length (Mb)	GC%
OX465281.1	1	258.85	38.5
OX465282.1	2	168.33	38.5
OX465283.1	3	167.37	38.5
OX465284.1	4	118.71	39.0
OX465285.1	5	102.3	39.0
OX465286.1	6	26.59	40.0
OX465287.1	MT	0.02	23.0

The estimated Quality Value (QV) of the final assembly is 58.8 with *k*-mer completeness of 99.99%, and the assembly has a BUSCO v5.3.2 completeness of 96.1% (single = 95.3%, duplicated = 0.8%), using the diptera_odb10 reference set ( *n* = 3,285).

Metadata for specimens, barcode results, spectra estimates, sequencing runs, contaminants and pre-curation assembly statistics are given at https://links.tol.sanger.ac.uk/species/2867258.

## Methods

### Sample acquisition and nucleic acid extraction

*Thereva unica* specimens was collected using an aerial net from Loe Valley (River Cober), England, UK (latitude 50.09, longitude –5.29) on 2021-06-29. The specimens were collected by Martin Drake and identified by Chris Spilling (both of the Dipterists Forum) and preserved by dry freezing at –80°C. The sample with specimen ID NHMUK014537453 (ToLID idTheUnic2), a female, was used for DNA sequencing was, and the sample with specimen ID NHMUK014537421 (ToLID idTheUnic1), a male, was used for Hi-C sequencing.

The workflow for high molecular weight (HMW) DNA extraction at the Wellcome Sanger Institute (WSI) includes a sequence of core procedures: sample preparation; sample homogenisation, DNA extraction, fragmentation, and clean-up. The sample was prepared for DNA extraction in the Tree of Life core laboratory. The idTheUnic2 sample was weighed and dissected on dry ice ( Jay *et al.*, 2023). Tissue from the whole organism was homogenised using a PowerMasher II tissue disruptor ( Denton *et al.*, 2023a). HMW DNA was extracted in the WSI Scientific Operations core using the Automated MagAttract v2 protocol ( Oatley *et al.*, 2023). HMW DNA was sheared into an average fragment size of 12–20 kb in a Megaruptor 3 system with speed setting 31 ( Bates *et al.*, 2023). Sheared DNA was purified by solid-phase reversible immobilisation ( Strickland *et al.*, 2023): in brief, the method employs a 1.8X ratio of AMPure PB beads to sample to eliminate shorter fragments and concentrate the DNA. The concentration of the sheared and purified DNA was assessed using a Nanodrop spectrophotometer and Qubit Fluorometer and Qubit dsDNA High Sensitivity Assay kit. Fragment size distribution was evaluated by running the sample on the FemtoPulse system.

Protocols developed by the WSI Tree of Life core laboratory are available on protocols.io ( Denton *et al.*, 2023b).

### Sequencing

Pacific Biosciences HiFi circular consensus DNA sequencing libraries were constructed according to the manufacturers’ instructions. DNA sequencing was performed by the Scientific Operations core at the WSI on a Pacific Biosciences SEQUEL II instrument. Hi-C data were also generated from head and thorax tissue of idTheUnic1 using the Arima2 kit and sequenced on the Illumina NovaSeq 6000 instrument.

### Genome assembly, curation and evaluation

Assembly was carried out with Hifiasm ( Cheng *et al.*, 2021) and haplotypic duplication was identified and removed with purge_dups ( Guan *et al.*, 2020). The assembly was then scaffolded with Hi-C data ( Rao *et al.*, 2014) using YaHS ( Zhou *et al.*, 2023). The assembly was checked for contamination and corrected as described previously ( Howe *et al.*, 2021). Manual curation was performed using HiGlass ( Kerpedjiev *et al.*, 2018) and Pretext ( Harry, 2022). The mitochondrial genome was assembled using MitoHiFi ( Uliano-Silva *et al.*, 2023), which runs MitoFinder ( Allio *et al.*, 2020) or MITOS ( Bernt *et al.*, 2013) and uses these annotations to select the final mitochondrial contig and to ensure the general quality of the sequence.

A Hi-C map for the final assembly was produced using bwa-mem2 ( Vasimuddin *et al.*, 2019) in the Cooler file format ( Abdennur & Mirny, 2020). To assess the assembly metrics, the *k*-mer completeness and QV consensus quality values were calculated in Merqury ( Rhie *et al.*, 2020). This work was done using Nextflow ( Di Tommaso *et al.*, 2017) DSL2 pipelines “sanger-tol/readmapping” ( Surana *et al.*, 2023a) and “sanger-tol/genomenote” ( Surana *et al.*, 2023b). The genome was analysed within the BlobToolKit environment ( Challis *et al.*, 2020) and BUSCO scores ( Manni *et al.*, 2021; Simão *et al.*, 2015) were calculated.

Table 3 contains a list of relevant software tool versions and sources.

**Table 3.** Software tools: versions and sources.

Software tool	Version	Source
BlobToolKit	4.2.1	https://github.com/blobtoolkit/blobtoolkit
BUSCO	5.3.2	https://gitlab.com/ezlab/busco
Hifiasm	0.16.1-r375	https://github.com/chhylp123/hifiasm
HiGlass	1.11.6	https://github.com/higlass/higlass
Merqury	MerquryFK	https://github.com/thegenemyers/MERQURY.FK
MitoHiFi	2	https://github.com/marcelauliano/MitoHiFi
PretextView	0.2	https://github.com/wtsi-hpag/PretextView
purge_dups	1.2.3	https://github.com/dfguan/purge_dups
sanger-tol/genomenote	v1.0	https://github.com/sanger-tol/genomenote
sanger-tol/readmapping	1.1.0	https://github.com/sanger-tol/readmapping/tree/1.1.0
YaHS	yahs-1.1.91eebc2	https://github.com/c-zhou/yahs

### Wellcome Sanger Institute – Legal and Governance

The materials that have contributed to this genome note have been supplied by a Darwin Tree of Life Partner. The submission of materials by a Darwin Tree of Life Partner is subject to the **‘Darwin Tree of Life Project Sampling Code of Practice’**, which can be found in full on the Darwin Tree of Life website here. By agreeing with and signing up to the Sampling Code of Practice, the Darwin Tree of Life Partner agrees they will meet the legal and ethical requirements and standards set out within this document in respect of all samples acquired for, and supplied to, the Darwin Tree of Life Project. 

Further, the Wellcome Sanger Institute employs a process whereby due diligence is carried out proportionate to the nature of the materials themselves, and the circumstances under which they have been/are to be collected and provided for use. The purpose of this is to address and mitigate any potential legal and/or ethical implications of receipt and use of the materials as part of the research project, and to ensure that in doing so we align with best practice wherever possible. The overarching areas of consideration are:

•   Ethical review of provenance and sourcing of the material

•   Legality of collection, transfer and use (national and international) 

Each transfer of samples is further undertaken according to a Research Collaboration Agreement or Material Transfer Agreement entered into by the Darwin Tree of Life Partner, Genome Research Limited (operating as the Wellcome Sanger Institute), and in some circumstances other Darwin Tree of Life collaborators.

## Data Availability

European Nucleotide Archive: *Thereva unica*. Accession number PRJEB57101; https://identifiers.org/ena.embl/PRJEB57101 ( Wellcome Sanger Institute, 2023). The genome sequence is released openly for reuse. The *Thereva unica* genome sequencing initiative is part of the Darwin Tree of Life (DToL) project. All raw sequence data and the assembly have been deposited in INSDC databases. The genome will be annotated using available RNA-Seq data and presented through the Ensembl pipeline at the European Bioinformatics Institute. Raw data and assembly accession identifiers are reported in Table 1.

## Author information

Members of the Natural History Museum Genome Acquisition Lab are listed here: https://doi.org/10.5281/zenodo.7139035.

Members of the Darwin Tree of Life Barcoding collective are listed here: https://doi.org/10.5281/zenodo.4893703.

Members of the Wellcome Sanger Institute Tree of Life Management, Samples and Laboratory team are listed here: https://doi.org/10.5281/zenodo.10066175.

Members of Wellcome Sanger Institute Scientific Operations: Sequencing Operations are listed here: https://doi.org/10.5281/zenodo.10043364.

Members of the Wellcome Sanger Institute Tree of Life Core Informatics team are listed here: https://doi.org/10.5281/zenodo.10066637.

Members of the Tree of Life Core Informatics collective are listed here: https://doi.org/10.5281/zenodo.5013541.

Members of the Darwin Tree of Life Consortium are listed here: https://doi.org/10.5281/zenodo.4783558.
