# Supplementary material for: The genome sequence of a stiletto fly, Thereva unica (Harris, 1780)
Source: Wellcome Open Res. 2024 Feb 12;9:27. [Version 1] doi: 10.12688/wellcomeopenres.20828.1 (PMC11099511; doi:10.12688/wellcomeopenres.20828.1)
Supplement: Peer review history

# Reviewer report: Reviewer response for version 1

**DOI:** 10.21956/wellcomeopenres.23047.r78800

**Published:** 2024-05-16

**Authors:** Alex Makunin

**Affiliations:** 1 Wellcome Sanger Institute, Hinxton, England, UK

The data note by Drake *et al.* presents the first reference genome assembly for the representative species stiletto fly family, *Thereva unica*. The report follows the standard structure for DToL project genome notes and it looks comprehensive and cohesive. 

- Interesting Hi-C patterns resembling inversions can be seen in chromosome 3 (FIgure 5).
- Although the sex chromosome was not identified at this time, the *Thereva nobilitata* genome (GCA_963855945.1, released by DToL a few months later) suggested X chromosome is the smallest chromosome based on synteny with idTolCing1 ( *Tolmerus cingulatus*) representing the same subfamily Asiloidea. Synteny between the two *Thereva* species genomes indicate chromosome 6 of *Thereva unica* as X chromosome. Interestingly, this chromosome also has slightly elevated coverage and GC content (Figure 3).

Are sufficient details of methods and materials provided to allow replication by others?

Yes

Is the rationale for creating the dataset(s) clearly described?

Yes

Are the datasets clearly presented in a useable and accessible format?

Yes

Are the protocols appropriate and is the work technically sound?

Yes

Reviewer Expertise:

comparative genomics

I confirm that I have read this submission and believe that I have an appropriate level of expertise to confirm that it is of an acceptable scientific standard.

# Reviewer report: Reviewer response for version 1

**DOI:** 10.21956/wellcomeopenres.23047.r78805

**Published:** 2024-05-16

**Authors:** Benoit Nabholz

**Affiliations:** 1 Universite de Montpellier, Montpellier, Occitanie, France

The manuscript presents the genome of a *Therevidae* fly, *Thereva unica*, produced by the Darwin Tree of Life project (DToL). Once again, I applaud the project for rapidly releasing all the information and data transparently. This project is a model of open science and should set the standard for consortium genome projects. This is the first genome published for the family (as opposed to the >500 assemblies currently available for *Drosophila*).

 I have very few comments. I have check all the links and all the data is available. The genome is chromosome scale.

 Here are my minor comments :

- The coverage of long reads is somewhat lower compared to other genomes I have reviewed for the project, mostly butterflies. Here, the coverage is 36X, whereas it was above 45X for most of the other genome assemblies I have checked (except *Hydrotaea diabolus*, which is another fly). For instance, the coverage of the other unpublished Thereva genome exceeds 99X, as noted on the NCBI website : https://www.ncbi.nlm.nih.gov/datasets/genome/GCA_963855945.1/.
  I wonder if this lower coverage will eventually lead to assembly errors, and I'm a bit concerned about that. Maybe it is enough using HiFi technology?
-  The coverage information for Hi-C data should be provided.

Are sufficient details of methods and materials provided to allow replication by others?

Yes

Is the rationale for creating the dataset(s) clearly described?

Yes

Are the datasets clearly presented in a useable and accessible format?

Yes

Are the protocols appropriate and is the work technically sound?

Yes

Reviewer Expertise:

Population genomics, Evolutionary biology, Entomology

I confirm that I have read this submission and believe that I have an appropriate level of expertise to confirm that it is of an acceptable scientific standard.

# Reviewer report: Reviewer response for version 1

**DOI:** 10.21956/wellcomeopenres.23047.r82503

**Published:** 2024-05-10

**Authors:** Juan Du

**Affiliations:** 1 Department of Entomology and MOA Key Lab of Pest Monitoring and Green Management, College of Plant Protection, China Agricultural University, Beijing, China

The authors provided a clear research motivation when preparing the dataset, thoroughly explaining the ecological and taxonomic significance of sequencing the genome of *Thereva unica*. The experimental protocols are appropriate and technically sound, including methods for sample collection, DNA extraction, and sequencing, which ensure the rigor and reliability of the research. However, in terms of the presentation and accessibility of the dataset, although the data is publicly available, the completeness of chromosome mapping is slightly lacking, which might affect the usability of specific analyses. From the standpoint of a simple data note, the overall quality of the genome assembly is satisfactory, but the genome annotation results are not accessible via the provided links. Overall, this article performs well in many critical aspects, but there is still room for improvement in the clarity and ease of use of the data presentation.

 The data is accessible, but the completeness of chromosome mapping（92%） is slightly below the standard（95%）, the genome annotation results are not accessible via the provided links. which could affect usability for certain types of analyses.

Are sufficient details of methods and materials provided to allow replication by others?

Yes

Is the rationale for creating the dataset(s) clearly described?

Yes

Are the datasets clearly presented in a useable and accessible format?

Partly

Are the protocols appropriate and is the work technically sound?

Yes

Reviewer Expertise:

epigenetics

I confirm that I have read this submission and believe that I have an appropriate level of expertise to confirm that it is of an acceptable scientific standard.
